# Haplotype Phasing of Biallelic *WNT10B* Variants Using Long‐Read Sequencing in Split‐Hand/Foot Malformation Syndrome

**DOI:** 10.1111/cge.14706

**Published:** 2025-01-18

**Authors:** Jelena Pozojevic, Naseebullah Kakar, Henrike L. Sczakiel, Nathalie Kruse, Kristian Händler, Saranya Balachandran, Varun Sreenivasan, Martin A. Mensah, Malte Spielmann

**Affiliations:** ^1^ Institute of Human Genetics, University Medical Center Schleswig‐Holstein University of Lübeck & Kiel University Lübeck Germany; ^2^ Department of Biotechnology FLS&I, BUITEMS Quetta Pakistan; ^3^ Institute of Medical Genetics and Human Genetics, Charité‐Universitätsmedizin Berlin, Corporate Member of Freie Universität Berlin and Humboldt‐Universität Zu Berlin Berlin Germany; ^4^ BIH Biomedical Innovation Academy, Berlin Institute of Health at Charité‐Universitätsmedizin Berlin Berlin Germany; ^5^ RG Development and Disease Max Planck Institute for Molecular Genetics Berlin Germany; ^6^ DZHK (German Centre for Cardiovascular Research), partner Site Hamburg/Lübeck/Kiel Lübeck Germany

**Keywords:** haplotype phasing, long‐read sequencing, split‐hand/foot malformation, *WNT10B*

## Abstract

Split‐hand/foot malformation syndrome (SHFM) is a congenital limb malformation that is both clinically and genetically heterogeneous. Variants in *WNT10B* are known to cause an autosomal recessive form of SHFM. Here, we report a patient born to unrelated parents who was found to be a compound heterozygote for missense variants in *WNT10B*: c.994C>T, p.(Arg332Trp) and c.638T>G, p.(Phe213Cys). The variants were identified using long‐read PacBio sequencing, which enabled phasing and confirmed that they were located on different alleles. The maternally inherited variant p.(Arg332Trp) has been previously reported, whereas the paternally inherited variant p.(Phe213Cys) is novel and absent from the gnomAD database. Our findings highlight the utility of long‐read haplotype phasing, which provides valuable insights in determining the biallelic nature of variants in recessive disorders when parental DNA samples are unavailable.

## Graphical abstract

Haplotype determination and phasing of compound heterozygous variants in *WNT10B* (autosomal recessive inheritance) in a patient with split‐hand/foot malformation syndrome. The biallelic nature of the two variants was detected by long‐read sequencing and confirmed by Sanger sequencing.

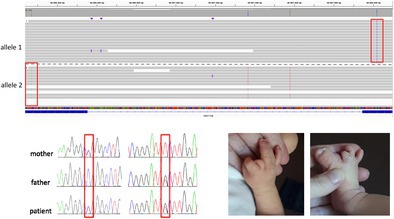

1

Whole exome sequencing (WES) is the standard technology in clinical genetics for detecting protein‐coding variants. Conversely, whole genome sequencing (WGS) can detect multiple genetic alterations, genome wide. Short‐read technologies, while highly accurate, cannot resolve repetitive and GC‐rich regions, but this problem can be overcome with long‐read sequencing that can resolve difficult sequences and provide epigenetic information.

Split‐hand/foot malformation (SHFM) is a congenital limb condition characterized by genetic heterogeneity, variable expression, and incomplete penetrance. Loci linked to SHFM include *TP63*, *DLX5*, *DLX6*, *FGFR1, MAP3K20*, *UBA2*, *BHLHA9*, and *WNT10B* [1]. Autosomal recessive, mainly homozygous *WNT10B* variants cause SHFM6 (OMIM #225300), while heterozygous variants cause dental anomalies [2, 3]. Here, we report a patient with SHFM caused by compound‐heterozygous *WNT10B* variants, detected using long‐read sequencing. This technology enabled us to phase the reads and prove the variants' biallelic nature.

The patient was born at 37 + 5 weeks with good overall health. The fontanelles, lymph nodes, and genitalia were unremarkable. However, skeletal limb abnormalities were noted. The right hand showed syndactyly of digits I + II and III + IV, with an extra finger between II + III. The left hand had five fingers with a skin bridge between III + IV. Both feet exhibited split malformations: the left foot lacked digit II, had a rudimentary digit III, and syndactyly of IV + V, while the right foot showed syndactyly of I + II and IV + V, with a rudimentary digit III (Figure 1A). Array CGH identified a maternally inherited intronic *TP63* deletion (chr3:189647416‐189650138; GRCh38), common in controls and therefore excluded as disease‐causing. Long‐read PacBio HiFi sequencing (average read length: 21081 bp) revealed two *WNT10B* variants: c.994C>T, p.(Arg332Trp) and c.638T>G, p.(Phe213Cys). Phasing (based on genome‐wide SNVs and indels) confirmed the variants were located on different alleles (Figure 1B). Sanger sequencing validated these findings, showing maternal inheritance of c.994C>T and paternal inheritance of c.638T>G (Figure 1C). The variant c.994C>T was previously reported as homozygous in a consanguineous family with SHFM [2], classified as pathogenic in ClinVar, with a CADD score of 32. According to ACMG criteria [4], it is pathogenic. The c.638T>G variant, absent in gnomAD, has a CADD score of 31 and is classified as likely pathogenic. AlphaFold [5] predictions showed that both variants altered WNT10B protein structure. p.(Phe213Cys) substituted a benzyl group with a thiol side chain, while p.(Arg332Trp) disrupted hydrogen bonds and replaced guanidino group with indole. Both variants were classified as pathogenic by the AlphaMissense pathogenicity score (Figure 1D,E).

**FIGURE 1:**
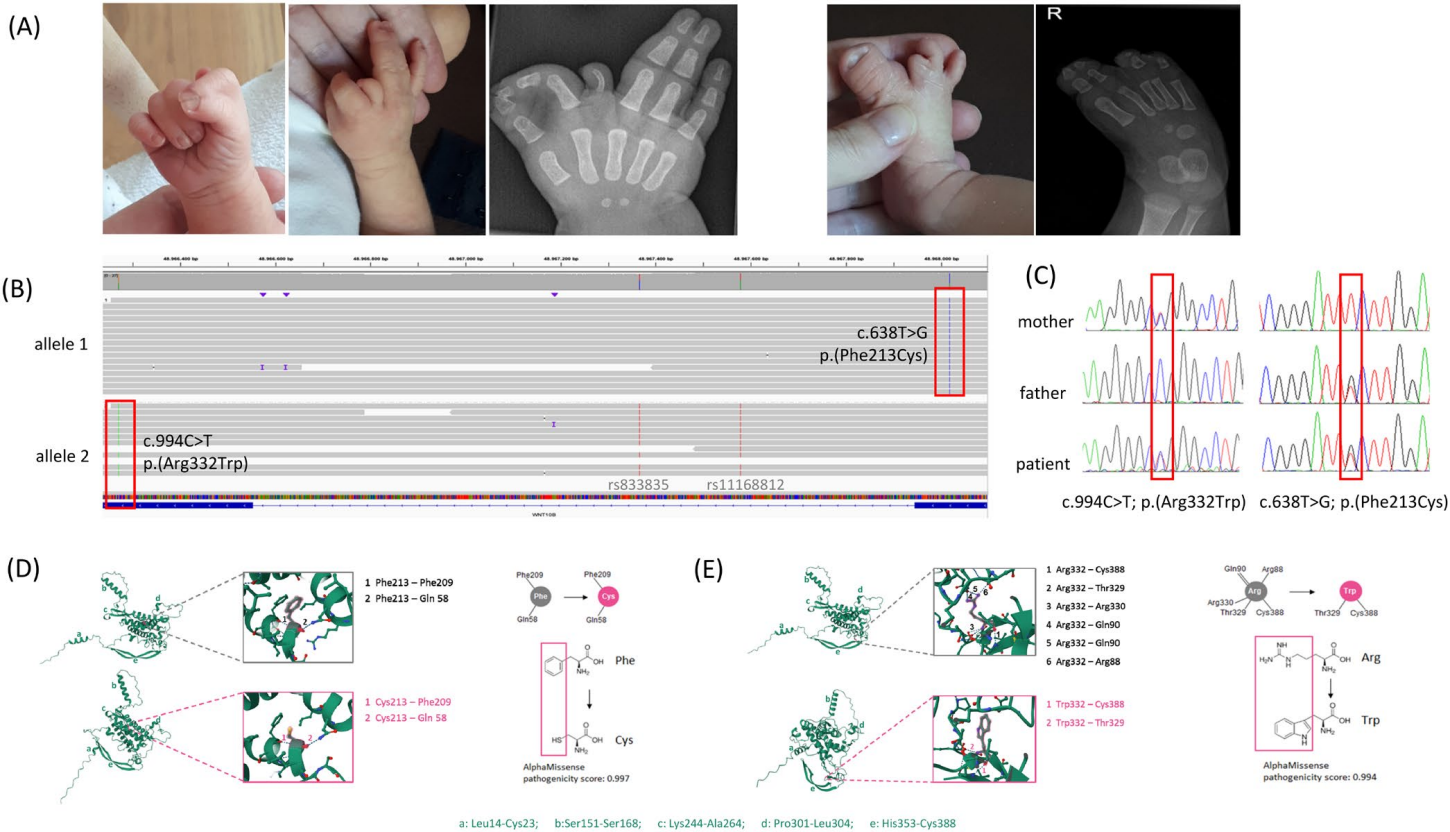
(A) Patient's phenotype. (B) Long‐read sequencing showing the biallelic variants and informative SNPs. (C) Sanger sequencing confirming the variants and their parent‐of‐origin. (D, E) *In silico* predictions of protein folding and hydrogen bonds for wild‐type versus mutant proteins, highlighting structural changes caused by each variant. Variants with AlphaMissense pathogenicity scores > 0.56 are classified as pathogenic.

Biallelic *WNT10B* variants cause autosomal recessive SHFM, and most cases are homozygous due to consanguinity, but few compound‐heterozygous variants have also been reported. Our patient was born to non‐consanguineous unaffected parents. After confirming that each of these variants was inherited from one parent, we sought to re‐evaluate the clinical status of the parents, since incomplete penetrance and dental anomalies were reported in heterozygous carriers, but they were unavailable.

Short read‐WES could have identified these variants at lower costs, but our work demonstrates the power of long‐reads in proving biallelic inheritance. This can be particularly informative when parental DNA is unavailable, when variants are *de novo*, or display mosaicism. Haplotype determination cannot be accurately done by short‐read sequencing and traditionally requires extensive experiments. In comparison, it is straightforward with long‐read sequencing to determine whether variants are in trans, supporting a recessive disease phenotype. Although this technology is typically used to resolve difficult sequences (structural variants, GC‐rich and repetitive regions, DNA methylation), we highlight its use in haplotype phasing, which can be critical in arriving at a molecular diagnosis. This study also expands the *WNT10B* mutational spectrum since c.638T>G has not been previously reported. As sequencing costs decrease, long‐read sequencing might become the preferred technology in clinical genetics, serving as a comprehensive diagnostic test, particularly in the absence of parental samples.

## Consent

Written informed consent was obtained.

## Conflicts of Interest

The authors declare no conflicts of interest.

### Peer Review

The peer review history for this article is available at https://www.webofscience.com/api/gateway/wos/peer‐review/10.1111/cge.14706.

## Data Availability

The data that support the findings of this study are available on request from the corresponding author. The data are not publicly available due to privacy or ethical restrictions.
